# Physical activity modification in youth with congenital heart disease: a comprehensive narrative review

**DOI:** 10.1038/s41390-020-01194-8

**Published:** 2020-10-13

**Authors:** Arend W. van Deutekom, Adam J. Lewandowski

**Affiliations:** 1 Division of Pediatric Cardiology, Department of Pediatrics, Erasmus MC-Sophia Children’s Hospital, Rotterdam, The Netherlands; 2 Oxford Cardiovascular Clinical Research Facility, Division of Cardiovascular Medicine, Radcliffe Department of Medicine, University of Oxford, Oxford, UK

## Abstract

**Abstract:**

Congenital heart disease (CHD) affects nearly 1% of births. As survival rates have dramatically improved, the majority of individuals with CHD now live into adulthood. As these patients age, they become prone to a large range of complications, such as chronic heart failure and acquired cardiovascular disease. Promotion of a healthy and active lifestyle from childhood onwards has been suggested as a sustainable and effective strategy to enhance cardiovascular health, improve quality of life and reduce immediate and long-term risk in people with CHD. Well-established physical activity consensus statements for youth with CHD have now been published. In this article, we review how increasing physical activity in youth with CHD may offer immediate and long-term cardiovascular benefits, what is known about physical activity in children with CHD, describe the unique factors that contribute to achieving sufficient and insufficient physical activity levels and summarize the evidence of trials on physical activity promotion in youth with CHD. Furthermore, we discuss some of the challenges that need to be addressed by further research regarding the optimal strategy, timing and format of physical activity intervention programmes in children and adolescents with CHD.

**Impact:**

- Congenital heart disease (CHD) affects nearly 1% of births, with the majority of individuals with CHD now living into adulthood due to improved survival. As CHD patients age, they become prone to a large range of cardiovascular complications.
- This article discusses how and why increasing physical activity in youth with CHD may offer immediate and long-term cardiovascular benefits, the barriers to achieving sufficient physical activity levels and the evidence from trials on physical activity promotion in youth with CHD.
- The optimal strategy, timing and format of physical activity intervention programmes in children and adolescents with CHD are discussed.

## Introduction

Major advances in the fields of paediatric cardiology and cardiac surgery over recent decades have dramatically improved the survival of patients with congenital heart disease (CHD). Currently over 90% of children who are born with CHD are expected to survive into adulthood.^1^ As these patients age, they are prone to long-term complications and comorbidities. For example, chronic heart failure is known to occur in a quarter of CHD patients by the age of 30 years, with the incidence increasing with age.^2^ CHD patients are also at increased risk for acquired cardiovascular diseases, such as hypertension, myocardial infarction and stroke, as approximately 80% of all young adults with CHD have at least one cardiovascular risk factor.^3^ These acquired cardiovascular diseases may further interact with arrhythmias or heart failure, worsening morbidity and mortality risk. Consequently, care for CHD patients has shifted from managing short-term survival to having the best possible outcome in terms of long-term physical health, development and well-being.

Promotion of an active lifestyle is increasingly recognized as pivotal for optimizing long-term health in CHD patients. In populations of patients with CHD, regular physical activity (PA) was shown to improve the features or delay the progression of systolic and diastolic heart failure, pulmonary hypertension and coronary artery disease.^4,5^ Exercise increases musculoskeletal strength and aerobic fitness, reduces cardiovascular and metabolic disease risk and event rates, increases healthy behaviours and promotes active lifestyles.^6^ In addition to the numerous physical health benefits, PA is associated with enhanced self-esteem, confidence, initiative, quality of life and social skills.^7^ Attempts to optimize PA levels early in life have the greatest potential for long-term health and well-being, because most healthy and active behaviours carry forward into adulthood.^8^ Therefore, every child with CHD should be encouraged to do sports and to participate in recreational PA during leisure time and at school. Unfortunately, most children with CHD are insufficiently physically active.

Several reviews have been written on exercise specifically in children with CHD and the effects of formal exercise interventions in these children.^9–15^ However, exercise and PA are two different constructs, with exercise being a subcategory of PA that is planned, structured, repetitive and purposeful in the sense that the improvement or maintenance of one or more components of physical fitness is the objective.^16^ PA also includes other activities, which involve bodily movement and are done as part of playing, working, active transportation, house chores and recreational activities. As such, it is important to recognize the significance of the full spectrum of PA for cardiovascular health, as activities of lighter intensity provide significant cardiovascular health benefits even if there are no changes in measures of exercise capacity.^17^ This aspect of PA in children with CHD is not currently well covered in the literature.

The primary purpose of this review is to evaluate the prevalence of physical (in)activity in children with CHD, to provide further insight into the unique risk factors of insufficient PA in children with CHD and to summarize the interventions that have been adopted to optimize activity levels in these children to date. As insufficient PA poses an additional health burden to patients with CHD, a thorough understanding of the prevalence and predictors of insufficient PA in children with CHD may have a significant long-term impact on the care of these patients.

## Methods

### Literature search

A comprehensive search was conducted using the PubMed database on May 19th, 2020. Search terms about CHD (e.g. cardiac defect, Fontan, etc.) were used independently and in combination with terms about PA (e.g. exercise, activity, etc.) and children (e.g. youth, child). The search was restricted by excluding non-relevant publication types (e.g. editorials, practice guidelines, biographies, etc.). Additionally, the reference lists of all selected articles and published reviews on this topic were screened for potentially relevant publications.

### Eligibility criteria

Studies were included if they met the following criteria: (i) the population included subjects aged <18 years with any form of CHD; (ii) the outcome was a measure of PA or sedentary behaviour. For intervention studies, we had the additional criterion that the intervention was aimed at improving PA levels.

### Data extraction

The following data were extracted from the studies that met our inclusion criteria: (i) general characteristics of the article (author’s name, publication year); (ii) study population (number, percentage female, age, type of CHD); (iii) inclusion criteria; (iv) exercise intervention (duration, intensity, type); (v) type of PA or sedentary behaviour studied; (vi) timing and method of measurement (objective or self-report); and (v) relevant results.

### Quality assessment

The two authors of this paper independently assessed the methodological quality of included studies, using a ten-item modified scoring system to reflect favourable design, reporting, as well as attrition and compliance (Supplementary Table 2).^18–20^ For each item, a score of 1 was allocated if the study met the criteria for that subcategory. Attrition and compliance criteria were based on standard guidelines for exercise intervention trials.^20^ Scoring for the intervention length and cohort size for the intervention group were determined using the median across studies. Summed scores between 0 and 4 were given an overall quality rating of ‘low’, 5–7 were scored as ‘moderate’, and 8–10 scored as ‘high’.

## PA and sedentary behaviour in children with CHD

There is a general belief that children with CHD have low levels of PA compared to their healthy peers.^21^ This is based on early studies that assessed PA levels in children with severe types of CHD >25 years ago.^22^ Since then, at least 18 articles have been published that assess the level of PA in children with CHD relative to a healthy population. Details of these observational studies and their findings with regard to PA are shown in Supplementary Table 1. Most studies enrolled adolescent patients, four studies enrolled younger school-aged children^22–25^ and one focussed on preschoolers.^26^ The number of patients with CHD included in the studies ranged from 7^23^ to 316,^27^ and the proportion of female subjects ranged from 32^28^ to 76%.^29^ The subjects had many different cardiac diagnoses in most studies. Three studies also included cardiac transplant recipients.^25,30,31^ Three studies only included subjects with Fontan circulation.^32–34^ In 14 of the studies, there was a control group of healthy age-matched children, while in the remaining 4 studies, PA levels were compared to previously published normative data from healthy cohorts.^30–32,35^ Different methods have been applied in assessing the daily levels of PA, including self- and parent-reported questionnaires and objective measures. The method of objective measurements was usually accelerometry. Heart rate monitors^24^ and the double labelled water method^23^ were used in one study each. All studies reported PA level, expressed as time in PA, exercise frequency, score on a PA scale or expended energy. In addition, eight studies described the proportion of individuals meeting national or international PA recommendations,^24,26,28,29,31,34,36,37^ while six studies also reported on the time spent sedentary.^26,29,31,33,34,37^

In 9 of the 18 (50%) studies, there were no statistically significant differences in PA level between children with CHD and age-matched healthy peers.^23,26–31,34^ Of the remaining nine studies, four reported that children with CHD were generally more inactive than the control group^22,24,38^ or than normative data.^35^ The other studies were ambiguous or found diminished PA only in subgroups. For example, two studies found that boys with CHD had lower accelerometry-assessed PA levels compared with healthy boys, while there were no differences in girls.^39,40^ In contrast, a study in 147 adolescents with a Fontan circulation found that, in girls, PA levels were markedly below normative data from healthy children, while boys were relatively unaffected.^32^ Interestingly, Arvidsson et al. found that CHD was only associated with lower PA levels in girls with CHD aged 9–11 years, not in older girls or boys, compared to age- and sex-matched peers.^36^ The method of measurement could explain part of the apparent discrepancies between the studies. Five^22,33,35,37,38,40^ out of eight^25,27,30^ studies with self- or parent-reported outcomes found significantly lower PA levels in children with CHD. In contrast, five of the nine studies with accelerometer-assessed PA found no differences between cases and controls,^26,28,29,31,33^ and three found lower PA levels only in subgroups of patients.^36,39,40^ The only accelerometer study that described a general lower PA level in children with CHD had no control group but compared the data to normative data from healthy children and adolescents participating in the Amherst Health and Activity Study.^32^ Of particular interest is the study by Hedlund et al. that assessed PA levels in adolescent Fontan patients by questionnaire and accelerometry simultaneously. The authors found that adolescents with CHD reported less time in physical exercise and with less intensity than healthy controls. However, the accelerometer-assessed activity level and the amount of time in moderate-to-vigorous PA did not differ between adolescents with CHD and controls.^33^

The proportion of children with CHD reaching the prevailing national or international recommendations of minimum PA (at least 60 min a day for most countries) varied between studies, ranging from 7^31^ to 76%.^28^ Six out of eight studies found that the proportion of children reaching these recommendations did not differ between children with CHD and either a control group^26,28,34,36^ or regional estimates.^31,37^ One study reported that 19 and 31% of the children after transposition of the great arteries and atrial septal defect/ventricular septal defect repair, respectively, reached national PA recommendations; significantly less than 57% of controls. However, Ewalt et al. found that a third of children with CHD were active for >60 min/day, which was a significantly larger proportion than the 4.8% of controls.^29^ The six studies that report on the sedentary time of children with CHD are in agreement that these children spent the majority of time sedentary, up to 74% of the monitored time.^34^ However, four studies found no differences between children with CHD and controls,^29,33,34^ two studies had no control group or normative data for comparison^31,37^ and one study actually reported less sedentary time in children with Fontan than healthy controls.^33^

Taken together, most of the studies on PA level in children with CHD—especially those using objective outcome measures—suggest that children with CHD have similar activity levels as their healthy peers and are not more sedentary. Although this might seem encouraging, the results need to be viewed in the context of alarmingly low levels of PA in children in general. Most recent estimates from a pooled analysis of 1.6 million adolescents from 146 countries, territories and areas globally showed that 81% of adolescents did not reach the recommended 60 min of moderate-intensity PA per day.^41^

## Correlates of PA

Understanding why children with CHD have an active or inactive lifestyle contributes to evidence-based planning of PA interventions. In doing so, effective programmes can be created to target factors known to cause inactivity or sedentary behaviour. In healthy children, the correlates of activity levels are multifactorial and include factors that are biological (sex, ethnicity, genetic predisposition), psychological (attitudes, motivation), sociocultural (parental and peer support, encouragement, social norms) and environmental (access to opportunities and facilities).^42,43^ Besides these traditional correlates, there are unique factors in children with CHD that may contribute to the development of an inactive lifestyle: cardiac capacity, formal exercise restrictions, parental overprotection, and perceived self-efficacy.

### Cardiac capacity

The normal haemodynamic adaptions to exercise may be, in a variety of ways and to a variable extent, adversely affected by CHD.^44^ For instance, patients with a Fontan circulation lack a functional right ventricle. During exercise, they cannot increase their pulmonary blood flow and pressures normally, which leads to insufficient left ventricular preload and systemic blood flow. In addition, they often have a lower arterial oxygen saturation due to residual shunting and a blunted heart rate response to exercise. Patients with tetralogy of Fallot often have abnormalities of their pulmonary vasculature and therefore may be unable to reduce their pulmonary vascular resistance normally. In addition, they may have significant residual haemodynamic abnormalities, such as severe pulmonary regurgitation and right ventricular dysfunction.^44^ Formal exercise testing in both patient groups has repeatedly shown that exercise capacity is markedly reduced, up to 50% in children with Fontan.^45,46^ Therefore, it is assumed that cardiac function would be strongly predictive of level of PA in these children.^27,47^ However, studies on this topic are conflicting. In a relatively large study in 147 children with Fontan, McCrindle et al. found that accelerometer-assessed PA was not related to medical history or maximum rate of oxygen consumption (VO_2_), VO_2_ at anaerobic threshold or maximum work rate during exercise testing.^32^ In a study with patients with a Fontan circulation, including 28 children below the age of 14 years, daily activity patterns were normal and there was no association with exercise capacity in the paediatric subgroup.^48^ Both Lunt et al. and Bar-Mor et al. found that disease severity did not predict self-reported level of sport or PA in their respective cohorts of adolescents with a range of CHD.^30,49^ In contrast, Ray et al. found a trend towards an inverse association of severity of CHD with the amount of PA in a similar cohort, although it was non-significant, possibly due to small sample size.^35^ Furthermore, in a large accelerometer study of 162 children with a full range of CHD diagnoses, the authors found that severity of heart disease and Fontan circulation were associated with not meeting the World Health Organization criteria for sufficient PA.^28^

### Exercise restrictions

Formerly, exercise restrictions placed by physicians were common practice in children and adolescents with CHD, due to fears of the possible proarrhythmic potential of exercise. Currently, exercise is considered safe and beneficial for almost all patient groups, but there remains hesitancy among physicians regarding the safety and efficacy of regular exercise in children with complex CHD.^50,51^ For example, Swan and Hillis found in a survey of 99 young adults who attended a paediatric and adult congenital outpatient clinic that relatively few patients were encouraged to exercise (19%) by their physician and PA advice was more often prohibitive (30%), even though the majority of patients had simple and haemodynamically non-significant lesions.^52^ Dean et al. reported that 40 of the 177 (22.5%) adolescents and young adults with CHD found themselves restricted from at least one sport by their doctor.^53^ Lunt et al. found that exercise advice by the physician had little influence on the PA level of adolescents with CHD.^30^ However, the consequences of physician-imposed PA restriction can be profound, as demonstrated by Stefan et al.^54^ They found that formal exercise restriction was the strongest predictor of being overweight or obese at 8.4 years follow-up in children with CHD, more so than exercise intolerance per se. Even children who were of healthy weight at baseline had a higher risk of becoming obese over time if their practitioners restricted their activity.

### Overprotection

Exercise restriction may also be imposed by their parent, educators or sports coach, rather than suggested by the child’s practitioner. In a landmark study, Bergman and Stamm reported that parents of children who have been evaluated for ‘innocent’ heart murmurs in the past often stated that ‘something was wrong with the heart’ of their child.^55^ In this cohort of 75 children, 30 children (40%) were restricted in their PA engagement by parents and teachers, sometimes despite a physician’s reassurance, and 15 children (20%) stated that their families worried about their hearts and that they were ‘treated differently’. Decades later, similar parental fears were found in children with severe CHD. In a group of 26 children with surgically palliated complex CHD, the majority of parents thought their child was unable to walk a distance of >100 yards, while formal exercise testing suggested that most of the group had only moderate functional disability.^22^ Furthermore, in the multicentre Paediatric Heart Network Fontan Study, parents perceived that their children were more impaired in their physical functioning than the children perceived themselves.^56^ However, more recent research does not support these findings. In a study in Korean adolescents with complex CHD, parental overprotection was not correlated with self-reported PA or sedentary behaviour level.^57^ Lunt et al. found that only 4 out of 153 adolescents with CHD reported that their parents do not encourage or help them as being a barrier to their activity; a smaller proportion than in a healthy reference group.^30^ Of note, parents helped in the survey, which may have affected the results. A study in young adults with CHD specifically surveyed about parental concerns regarding participation in sport and exercise.^52^ Only 3.3% of the patients felt their parents had inappropriate concern and 43% felt their parents’ concern was appropriate. However, 16% of patients had health fears related to their heart condition themselves, thus it is plausible that restrictive attitudes of parents and patients co-exist.

### Perceived self-efficacy

Youth with CHD sometimes display poor self-esteem and self-efficacy towards PA, especially in patients with more complex CHD. For example, in the study of Bar-Mor et al., self-efficacy was found to be an important determinant of PA participation, while objective severity of the cardiac malformation was not.^49^ Moola et al. quantitatively explored perceptions towards PA and sport in the lives of youth with CHD and found that a large proportion of the 13 adolescents displayed low self-efficacy towards sport.^58^ This was driven by unpleasant social interactions and comparisons with athletic healthy peers, and led to a decreased value ascribed to PA and sport. Kwon et al. also found that lower self-efficacy was associated with a lower engagement in PA in adolescents with complex CHD,^57^ but the findings of McCrindle et al. were more ambiguous.^32^ In their cohort of 147 paediatric Fontan patients, lower measured PA levels were significantly related to lower perceived general health but not to self-esteem or social impact of physical limitations. In a cohort of children with less severe CHD (operated with no residual disease), self-efficacy was an important predictor of PA, explaining almost a quarter of the variability in PA level.^38^ However, in a study among 10–14-year-olds with mild to moderate or surgically repaired CHD, self-efficacy scores were comparable to those previously published for healthy adolescents.^37^

Closely related to self-efficacy is the anxiety or fears towards PA that patients with CHD may have. For example, a study conducted in the United Kingdom found that 12% of children with CHD overestimated the extent of their exercise restrictions, needlessly avoiding PA participation.^59^ In addition, 30% of 316 adolescents with repaired atrial septal defect, ventricular septal defect or tetralogy of Fallot perceived ‘cardiac symptoms’, but these were not related to cardiac diagnosis or the presence of residual lesions. These symptoms were predictive of a lower score for exercise behaviour, while diagnosis or residual lesions were not.^53^ In young adults with CHD, the most prevalent barriers to exercise were perceived symptoms, but these symptoms often occurred in the presence of non-haemodynamically significant lesions.^52^ The authors suggest that ‘normal’ symptoms indicative of deconditioning are misinterpreted as ‘pathological’ cardiac symptoms related to their CHD and that anxiety or preoccupation with health may contribute.

In summary, behavioural factors appear to influence inactivity more than the severity of the heart condition or level of physical fitness. Structured PA approaches to let children experience success and improved physical functioning might increase self-efficacy and be a fruitful way to stimulate PA in this group. In addition, some children with mild or repaired CHD with no residual haemodynamic lesions perceive symptoms while exercising, which may be the result of deconditioning. This component of their disability should respond to interventions to improve PA. Although theoretically appealing, proof of this concept has been limited.

## Interventions to improve PA

As discussed, there are many studies on the effects of formal exercise interventions on exercise capacity in children with CHD, covered in several systematic reviews. However, there are only eight reported trials that assessed the effect of an intervention on general levels of PA in children with CHD.^47,60–66^ The characteristics of the studies are summarized in Table 1. The number of subjects with CHD included per study ranged from 25 to 168, and all trials but one^64^ enrolled adolescent patients. Two trials specifically focussed on children with Fontan circulation^62,64^ and one on children with either Fontan or tetralogy of Fallot.^60^ Five trials had a control group of children with CHD who did not receive the intervention, one trial used a group of healthy children who also received the training programme for comparison^62^ and two studies had no control group.^64,65^ Interventions across trials were heterogeneous, with the duration of the intervention ranging from 3 days^65^ to 1 year.^63,64^ Most studies performed standardized, supervised exercise programmes close to the participants’ home. Of the other studies, one trial evaluated the effect of a sports camp,^65^ one trial used motivational interview style group sessions^66^ and one trial used a mobile application and individually tailored text messages to stimulate PA.^63^ Three studies used a self-reported questionnaire to measure PA levels,^47,62,65^ and five used objective measures. None of the studies assessed sedentary behaviour.

**Table 1:** Characteristics of studies on the effect of an intervention aimed to increase physical activity in children with congenital heart disease.

Study	Subjects			Exercise intervention	Outcome			Results	Quality^a^
	*n*; age (mean ± SD), % girls		Cardiac diagnosis (*n*); additional inclusion criteria (if applicable)						
	Intervention	Control			Measure	Method of measurement	Timing of measurement		
Dulfer et al.^67^ and Duppen et al.^60^	56; 15 ± 3 years, 24%	37; 16 ± 3 years, 30%	ToF (47), Fontan (44)	12-week standardized aerobic exercise training programme	Physical activity; active leisure time	5-day accelerometry; self-report questionnaire	Directly after intervention	Exercise training did not change time spent in PA or self-reported active leisure time in either ToF or Fontan patients	High
Fredriksen et al.^61^	55; 12.4 ± 1.5 years, 45%	38, age n/a, 50%	LVOTO (20), TGA (17), ASD/VSD (16), ToF (16), RVOTO (9), Fontan (6), other (9); physical fitness equal or poorer than peers	2-week training programme in a rehabilitation sport centre or a 5-month training programme near their own home twice a week	Exercise time	1–2-week activity tracker	1–2 weeks after intervention	Exercise time increased similarly between groups from 677 ± 151 to 708 ± 152 s/day in parallel with increase in peak oxygen uptake and ventilation	Low
Hedlund et al.^62^	30; 14.2 ± 3.2 years, 46.6%	25; 13.6 ± 3.5 years, 48.0%	Fontan	12-week, twice a week, 45 min, individualized endurance training programme near home	Exercise time	Self-report questionnaire	Directly after intervention, 1 year	In the intervention group, self-reported exercise time increased from 113.5 ± 66.1 at baseline to 168.3 ± 92.7 min/week. At 1-year follow-up, amount of exercise returned to baseline	Moderate
Klausen et al.^63^	81, 14.6 ± 1.3 years, 41.8% (of full cohort)	77, 14.6 ± 1.2 years, 41.8% (of full cohort)	CoA (52), TGA (35), ToF (21), AVSD (9) DORV (7), Fontan (6), TA (4), other (24)	52-week eHealth intervention delivering individually tailored text messages to encourage PA	Time in MVPA	6 day accelerometry, self-report questionnaire	Directly after intervention	Patients in the intervention group spent 40.3 ± 21.8 min/day in MVPA compared to 41.3 ± 22.9 min/day for controls (not significantly different). Assessments of PA by questionnaire yielded similar results	High
Longmuir et al.^64^	61; 9.1 years [IQR 7.7–10.5], 41%	—	Fontan	12-month, weekly parent-lead, home-based intervention to enhance PA, motor skill, fitness, and activity attitudes	MVPA	7-day accelerometry	At the start of intervention, 6 months, 12 months (end of intervention), 24 months	MVPA increased significantly by 6 months, decreased at 12 months and then increased again to 36 ± 31 min/week (*p* = 0.04) above baseline at 24 months	Moderate
Moons et al.^65^	25; 12 years (IQR 10.5–13], 28%	—	Fontan (6), ToF (4), TGA (3), other (12)	3-day multisports camp for children with CHD	Habitual PA	Self-report questionnaire	3 months after intervention	No differences observed in habitual PA scores	Low
Morrison et al.^66^	72; 15.3 ± 2.2 years, 33.3%	71; 15.9 ± 2.2 years, 46.5%	Acyanotic corrected CHD (61), minor CHD (39), cyanotic corrected (30), cyanotic palliated (13)	6-month, monthly motivational interview-style group sessions	MVPA, % meeting national UK PA recommendations (>60 min/day MVPA)	7-day accelerometry	Directly after intervention	Significant increase in MVPA from 28.4 ± 20.1 to 57.2 ± 32.2 min/day (*p* < 0.001) for the intervention group while MVPA remained the same in controls. Number of patients meeting the PA recommendation doubled from 14 to 29	High
Rhodes et al.^47^	15; 11.9 ± 2.2 years, 26.7%	18; 12.1 ± 2.5 years, 22.2%	Complex CHD; peak work rate and/or peak VO_2_ < 80% of predicted	12-week, twice a week, 1 h exercise sessions	Exercise frequency	Self-report questionnaire	1 year after intervention	Patients in the intervention group reported they exercised more frequently than the year before; the control subjects did not (*p* = 0.07 for between-group difference)	Low

*ASD* atrial septal defect, *AVSD* atrioventricular septal defect, *CHD* congenital heart disease, *CoA* coarctation of the aorta, *DORV* double outlet right ventricle, *LVOTO* left ventricular outflow tract obstruction, *MVPA* moderate-to-vigorous physical activity, *PA* physical activity, *RVOTO* right ventricular outflow tract obstruction, *TA* tricuspid atresia, *TGA* transposition of the great arteries, *ToF* tetralogy of Fallot, *VSD* ventricular septal defect.

^a^For quality scoring, see Supplementary Table 2.

Two trials with objective PA assessment^61,66^ and two trials with subjective outcome data^47,62^ reported a significant increase in PA levels directly after completion of the intervention. Interestingly, in one of these studies, a control group who only received information about the benefits of PA also increased in exercise time.^61^ This suggests that providing accurate health information regarding the importance of PA and providing reassurance that PA is harmless for their heart might be sufficient to increase PA in children with CHD. The other four studies found no effect of the intervention on subjects’ PA level.

Long-term sustainability of training programmes is an important goal, but data are limited. Only two studies described longer-term effects, measured 1 year after completion of the intervention. Hedlund et al. found that self-reported exercise time increased directly after 12-week endurance training but returned to baseline at 1-year follow-up.^62^ In contrast, Longmuir et al. implemented a 12-month cardiac rehabilitation programme emphasizing PA and healthy lifestyle behaviours among a cohort of 61 children after Fontan palliation.^64^ They found that time in moderate-to-vigorous PA increased significantly by 6 months after starting the intervention, was decreased at the end of the 12-month programme and then increased again to 36 ± 31 min/week (*p* = 0.04) above baseline.

Taken together, this evidence shows that although exercise interventions might be a safe and effective method to increase PA in youth with CHD, the body of knowledge is very limited, especially in preadolescent children. Methodological issues, inconsistent and short-term results, as well as a low number of participants, are still major challenges (see Supplementary Table 2). Furthermore, adherence to the training programme is an important issue, with dropout and non-compliance rates up to 50%. These low levels of adherence may indicate that the exercise intervention is perceived as an optional service, where dropout can be assumed to have little medical consequences, or may reflect low satisfaction or success rates.

The reasons for not participating, withdrawal and non-compliance to exercise interventions have been explored by Dulfer et al.^67^ They concluded that logistical issues and time constraints of the parents were important barriers to participation. Second, adherence to the intervention was strongly related to fun that adolescents had during the exercise sessions. Third, anxieties and concerns regarding sports were present in both adolescents and parents, which contributed to reluctance to participate and withdraw. Therefore, the authors emphasize that future exercise interventions should have a more fun, age-attuned character, possibly including ‘game-like’ elements, and should address unanswered questions and unrealistic fears and attitudes towards PA.^68^

## Future in exercise interventions: ‘wearables’ and ‘gamification’?

New technological advancements provide exciting novel opportunities to overcome the challenges in exercise interventions in children with CHD. The rapidly expanding availability of mobile technologies such as ‘wearables’ and apps provide a platform to monitor daily health behaviours and to deploy interventions in a home-based context. This platform could use game design elements, referred to as ‘gamification’, for example, by using activity level (e.g. steps taken, energy expended) to score points, reach goals or complete challenges.^69,70^ The advantages compared to traditional PA measurement devices could be manifold: improved adherence to device wear protocols through ‘gamified’ or individualized approaches; greater ability to access PA data remotely to incorporate continuous PA monitoring, counselling and promotion into the intervention; and the possibility to provide real-time feedback to the child (and their parents), so that a wearer can be aware of their PA level, exertion and physical response during exercise.^70^ Although there is no experience with these technological advancements in children with CHD, it has been shown in adults post-myocardial infarction that smartphone-based technology improved uptake, adherence and completion of a rehabilitation programme.^71^ In children with obesity, gamified wearables have been shown to help children increase their PA level and lose weight.^72^ Albeit theoretically appealing, the question remains whether gamified interventions are effective in driving behaviour change, health and well-being in children with CHD and, more specifically, whether they promote long-term healthy behaviours.

## Current clinical practice and recommendations for improvement

Although emphasis is placed on lifestyle medicine in adult cardiology, it is not often a focus of the paediatric cardiology outpatient visit. In a survey to evaluate counselling on modifiable cardiovascular risk factors, paediatric cardiologists answered that they believed that promotion of cardiovascular health was a more appropriate role for primary care providers than for paediatric cardiologists.^73^ In another study on exercise advice in paediatric cardiac clinics, clinicians indicated that they do not have the knowledge, skills, resources or time needed to implement the extensive literature on PA promotion or to counsel patients about PA.^74^ In line with this, in a study of the care of overweight children with heart disease at two major paediatric heart centres, practitioners did not document lifestyle and weight counselling in >85% of children.^75^

These practices contrast sharply with international scientific statements on the importance of an active lifestyle for future cardiovascular health in children with CHD.^8,21,51^ These statements emphasize the need for a proactive promotion of PA by health professionals. A workgroup of the Association for European Paediatric and Congenital Cardiology has developed lesion-specific recommendations for exercise participation and assessment for children with CHD.^51^ They stress that almost all children with CHD can engage in sporting activities with no or minimal restrictions and should be encouraged to reach published recommendations of 60 min of moderate intensity exercise daily. In addition, children should perform <2 h a day of sedentary activities. PA counselling should be part of every patient interaction, and clinicians should provide patients, parents and primary care providers with recommendations in writing. Indeed, written instructions have been found to be more effective in increasing PA levels among sedentary patients than verbal communication alone.^76,77^ Although these guidelines are largely consensus based and extrapolated from PA recommendations for healthy children, they warrant a paradigm shift in all of clinical care by enhancing knowledge, understanding and appreciation of the multiple benefits of regular PA for every child, for every ability and at all ages.

## Conclusions

Given that most children with CHD now survive into adulthood and enjoy near-optimal functioning and well-being, care for these children now includes the prevention of long-term complications and comorbidities. The majority of children with CHD have inactive lifestyles, marked by low PA levels and excessive sedentary behaviour. In addition to traditional risk factors for inactivity, unique risk factors such as formal exercise restrictions, parental overprotection and fear to exercise may mediate the relationship between CHD and low PA levels in children. Interventions to stimulate exercise and other PA engagements in children with CHD have demonstrated that regular exercise is safe and there is some evidence that it increases activity level in children with CHD in the short term, although the evidence base is limited. There is much uncertainty about the optimal strategy, timing and format (home-based, new technology) of exercise interventions, as well as the long-term effects of these programmes on PA and cardiovascular health, which should be the focus of future research. Clinicians looking after children with CHD need to strongly advocate the importance of an active lifestyle based on published consensus statements for PA in youth with CHD, because children who develop strong patterns of healthy PA are likely to continue heart-healthy habits into adulthood.

## Supplementary information

Supplemental Tables
