# Supplementary material for: Physical activity modification in youth with congenital heart disease: a comprehensive narrative review
Source: Pediatr Res. 2020 Oct 13;89(7):1650–8. doi: 10.1038/s41390-020-01194-8 (PMC8249230; doi:10.1038/s41390-020-01194-8)
Supplement: Supplementary file 1 — Supplemental Tables [file 41390_2020_1194_MOESM1_ESM.doc]

| **Supplemental Table 1. Characteristics of observational studies on the level of physical activity in children with congenital heart disease** | | | | | | |
| --- | --- | --- | --- | --- | --- | --- |
| **Study (first author, year, reference)** | **Subjects** | | | **Outcome** | | **Results** |
|  | **N, age (mean±SD), % girls** | | **Cardiac diagnosis (n); notable additional inclusion criteria if applicable** |  |  |  |
|  | **Cases with CHD** | **Healthy controls** |  | **Measure** | **Method of measurement** |  |
| **Arvidsson, 2009(1)** | Two age groups:  I. 45, range 9-11y, 49%  II. 38, range 14-16y, 45% | Two age groups:  I. 87, range 9-11y, 53%  II. 73, range 14-16y, 49% | VSD (16), CoA (14), ASD (9), AoS (7), ToF (6), TGA (6), Fontan (3), other (22) | Total PA, type, frequency and duration of sport participation, % meeting PA recommendations from literature (60min/d MVPA) | 7d accelerometry; self-report questionnaire | The younger girls with CHD had lower PA levels than age-matched girls. No differences in PA levels between cases and controls were found in older girls or boys. No difference was found for any of the variables of self-reported sports participation. Four subjects (1 younger control girl, 1 younger patient boy, 2 younger control boys) met the recommended 60 min per day of MVPA. |
| **Barbour-Tuck, 2020(2)** | 32, 10.9±2.6y, 37.5% | 23, 11.7±2.5y, 43.5% | Fontan (7), ToF (5), cardiac transplant (4), TGA (3), septal defects (3), other (10) | Composite 5-point Likert score, with higher number indicating greater PA level | Self-report questionnaire | CHD and control groups were similar in PA score (3.05±0.71 and 3.35±0.69, respectively; p=0.12) |
| **Brudy, 2019(3)** | 162, 11.8±3.2y, 32% | 96, 10.9±3.8y, 51% | RVOTO (53), LVOTO (28), Fontan (25), isolated shunts (20), TGA (19), other (17) | Step count, MVPA, % meeting WHO’s PA recommendations (60min/d PA on weekly average) | 7d accelerometry | Compared to healthy controls, children with CHD had a slightly lower step count (10.206 ± 3.178 steps vs. 11.142±3.136 steps; p=0.04) but no lower MVPA (80.5±25.6min/d vs. 81.5±25.3; p=0.77). 75.9% of children with CHD achieved recommended minimal PA level, similar to controls (p=0.217). |
| **Casey, 1994(4)** | 26, 8.8±3.3y, % girls n/a | 26, 8.6±2.0y, % girls n/a | Palliated cyanotic CHD | Activity score | Parent-report questionnaire | There is a clear reduction in PA score in children with palliated cyanotic CHD compared with controls (data not shown). |
| **Chen, 2007(5)** | 316, 15.0±1.9y, 51.3% | 893, 14.5±2.0, 50.2% | VSD (104), ASD (58), ToF (30), multiple defects (67), other (57) | Exercise behaviour | Self-report questionnaire | There was no difference in exercise behaviour score between cases and controls (15.3±4.4 vs. 15.6±4.0, p=0.078). |
| **Ewalt, 2012(6)** | 21, 10.7±3.2y, 76% | 21, 10.8±3.2y, 76% | HLHS (4), CoA (4) VSD (2), MV prolapse (2), other (9) | Total PA level, PA intensity, sedentary behaviour, % meeting national USA PA recommendations (>60min/d PA). | 7d accelerometry | There were no differences in total PA (287.2±110.1 counts/30s/d for cases, 262.0±57.5 for controls, p=0.30), PA intensities or sedentary behaviours (398.7±106.5min/d vs. 405.7±89.9, p=0.712) between children with CHD and controls. 33.3% of children with CHD and 4.8% of controls met PA recommendations. |
| **Fredriksen, 2000(7)** | 125, 12.1±1.8y, 49.6% | 66, 12.0±1.2y, 59.1% | n/a | Total PA level | 7d accelerometry | Boys with CHD had a significantly lower PA level than control boys (p = 0.003), but no difference was found in girls (p = 0.76). |
| **Härtel, 2020(8)** | 11, age range 14-18y, 42.9% girl (of total intervention group of 21 Fontan patients including adults) | 6, age range 14-18y, 45.0% girl (of total control group of 20 controls including adults) | Fontan | Total PA level, sedentary behaviour, % meeting WHO’s PA recommendations | 5d accelerometry | Teenagers with CHD showed similar frequencies and intensities of PA and time spent sedentary compared to age-matched controls. Only 3 patients and 2 controls achieved the WHO recommended minimum of daily PA. |
| **Hedlund, 2016(9)** | 30; 14.2±3.2y, 46.6% | 25; 13.6±3.5y, 48.0% | Fontan | Time spent sedentary and in total, light, moderate and vigorous PA and in sedentary behaviour; self-reported time in and intensity of exercise. | 7d accelerometry, self-report questionnaire | Patients reported less time in exercise than controls (113.5±66.1 vs. 227.6±147.2 min/week, p < 0.001) and with less intensity. (13.0±2.1 vs. 14.3±1.9, p<0.05). However, accelerometry-assessed time in total PA and MVPA did not differ between the patients and controls. Patients spent less time in sedentary activity than the control (48.6±4.4% vs. 51.8±5.2%, P<0.05). |
| **Kao, 2009(10)** | 34, 10.5±1.0y, 50.0% | 34, 10.4±1.0y, 50.0% | VSD (16), ToF (11), ASD (7) | Total energy expenditure (TEE), level of total PA, level of MVPA | 3d accelerometry, Self-report questionnaire | The average TEE measured by both self-report and accelerometry and PA level measured by accelerometry were lower for boys with CHD than boys with no CHD. No differences in girls. Level of MVPA was similar between children with and without CHD for boys and girls. |
| **Leitch, 2000(11)** | 7, 5.7±0.5y, 57.1% | 10, 5.2±0.6y, 50.0% | ToF (4), Fontan (3) | Total and activity energy expenditure | Double labelled water method | Total and activity energy expenditure assessed were not significantly different between cases and controls. |
| **Lunt, 2003(12)** | 153, 14.6±1.8y, 38% | - | Mild CHD (e.g., ASD, VSD, PDA, CoA, n=110), Severe CHD (e.g., TGA, Fontan, cardiac transplant, n=43). | Total PA level, duration and frequency of PA. | Self-report questionnaire | Overall, adolescents with CHD are as physically active as expected from normative data from healthy children. However, compared to this reference, fewer are as vigorously active during either summer or winter, especially boys. |
| **Massin, 2006(13)** | I. 52, 9.8±1.8y,  26.9%;  II. 35, 9.1±2.0y, 37,1%. | 127, 10.0±2.3y, 48,8%. | I. TGA;  II. ASD (16), VSD (19); repair before the age of 3 years. | Total PA level, intensity of PA, % meeting national Belgian PA recommendations (>30 min/d of moderate PA and >20min/d of vigorous PA) | 4d heart rate monitor | Both children after TGA and children after ASD or VSD repair were significantly less active than the control group when considering moderate (25.3±12.9min/day, 26.2±11.7, 31.8±13.9 resp., p = 0.026) and vigorous activities (15.7±11.3, 16.2±9.1, 21.9±11.3, p = 0.006). Only 19% and 31%% of the children after TGA and ASD/VSD, respectively, reached national PA recommendation, versus 57% of controls (p=0.004). |
| **McCrindle, 2007(14)** | 147, median age 11.6y (range 1.5-16), 38%. | - | Fontan | Mean counts per minute, PA intensity | 7d accelerometry. | Time in moderate and vigorous activity was markedly below normative data from healthy children and adolescents participating in the Amherst Health and Activity Study at all ages, particularly in females. |
| **Moschovi, 2019(15)** | 76, 12.7±1.6y, 39.5% | 78, 12.5±1.0y, 48.7% | Simple complexity CHD (e.g., ASD, VSD, n=22), Moderate complexity CHD (e.g., ToF, SAS, n=48, Great complexity CHD (e.g., single ventricle, TGA, n=6). | Total PA level, PA intensity, average MET activity | Self-report questionnaire | Total activity level and average MET activity were lower for children with CHD than controls (MET 2.38±0.85 vs. 3.10±0.95, resp.). Children with CHD were more likely to engage in low PA. Differences between cases and controls were bigger for higher intensity of PA: controls had on average 3.18±2.06 30-minute blocks with a >6 МЕΤ vs. 1.8 ± 1.8 blocks for children with CHD (P<0.001). |
| **Ray, 2011(16, 17)** | 84, 12±1.4y, 39.3% | - | Surgically treated CHD (34), complex or severe CHD (19), moderate CHD (18), mild CHD (13) | Mean PA score, time in PA, screen time, % meeting CDC PA recommendations (>60min/d PA) | Self-report questionnaire | Children had much lower PA scores than those published for healthy children (31.7±11.2 METs vs. 53.5, resp.) 38% of children reached recommended PA levels, comparable to statewide normative data (p= 0.48). Children reported <1hr/day of watching TV or playing videogames in 45.3% and 60.7% of cases, respectively. |
| **Stone, 2015(18)** | 10, 4.0±0.9y, 40% | 10,4.2±1.0y, 40% | CoA (6), ToF (4) | Time in total, light, moderate, vigorous and very-vigorous PA, sedentary behaviour, % meeting national Canadian PA recommendations (60min/d MVPA) | 7d accelerometry | No significant differences were observed between patients and controls for total PA (p = 0.80), any of the PA intensities (p=0.71, 0.46, 0.43, and 0.45, resp.), or sedentary behaviour (p=0.38). 3 patients and 2 controls reached the recommended daily amount of PA. |
| **Voss, 2017(19)** | 90, 13.6±2.7y, 46% | - | Severe CHD (e.g., Fontan, TGA, n=29), mild CHD (e.g., ASD, mild PS, n=26), moderate CHD (e.g. CoA, ToF, n=26), cardiac transplant (9) | MVPA, sedentary behaviour, % meeting PA guidelines’ recommendations (≥60 min/d MVPA on at least 6d/week), sport participation | 7d accelerometry, self-report questionnaire | Mean MVPA was no different from normative data (49 vs. 50 min/day resp.). Median PA questionnaire score was 2.6 of 5 (IQR 2.1–3.1), which is somewhat lower than scores reported for healthy children. Adherence to PA guidelines was estimated at 8%, similar to national estimates (7%). |

AoS: aortic stenosis; ASD: atrial septal defect; AVSD: atrioventricular septal defect; CHD: congenital heart disease; CoA: coarctation of the aorta; DORV: double outlet right ventricle; HLHS: hypoplastic left heart syndrome; LVOTO: left ventricular outflow tract obstruction; MV: mitral valve; MVPA: moderate to vigorous physical activity; PA: physical activity; PS: pulmonary stenosis; RVOTO: right ventricular outflow tract obstruction; TA: tricuspid atresia; TGA: transposition of the great arteries; ToF: tetralogy of Fallot; VSD: ventricular septal defect

**Supplemental Table 2. Quality scoring of intervention studies aimed to increase physical activity in children with congenital heart disease**

|  | **Favourable design:** | | | | |  | **Reporting of:** | | |  | **Attrition/Compliance:** | | **Score** | **Quality** |
| --- | --- | --- | --- | --- | --- | --- | --- | --- | --- | --- | --- | --- | --- | --- |
| Publication: | Cohort Size | Control Group | Matching | Physical Activity Assessment | Intervention Length |  | Attrition | Compliance | Baseline Physical Activity |  | Attrition | Compliance |  |  |
| **Dulfer, 2014(20) & Duppen, 2015(21)** | 1 | 1 | 1 | 1 | 1 |  | 1 | 1 | 1 |  | 1 | 1 | **10** | **High** |
| **Fredriksen, 2000(22)** | 0 | 1 | 0 | 1 | 0 |  | 0 | 0 | 1 |  | 0 | 0 | **3** | **Low** |
| **Hedlund, 2018(23)** | 0 | 1 | 1 | 0 | 1 |  | 1 | 0 | 1 |  | 1 | 0 | **6** | **Moderate** |
| **Klausen, 2016(24)** | 1 | 1 | 1 | 1 | 1 |  | 1 | 1 | 1 |  | 0 | 0 | **8** | **High** |
| **Longmuir, 2013(25)** | 1 | 0 | 0 | 1 | 1 |  | 1 | 1 | 1 |  | 1 | 0 | **7** | **Moderate** |
| **Moons, 2006(26)** | 0 | 0 | 0 | 0 | 0 |  | 0 | 0 | 1 |  | 0 | 0 | **1** | **Low** |
| **Morrison, 2013(27)** | 1 | 1 | 0 | 1 | 1 |  | 1 | 1 | 1 |  | 0 | 1 | **8** | **High** |
| **Rhodes, 2006(28)** | 0 | 1 | 1 | 0 | 1 |  | 0 | 0 | 1 |  | 0 | 0 | **4** | **Low** |

Favourable design: Cohort Size (0 = n<56 in intervention arm, 1 = n≥56 in intervention arm); Control Group (0 = no control group, 1 = control group); Matching (0 = >10% sex difference and >1SD age difference between groups, 1 = ≤10% sex difference and ≤1SD age difference between groups); Physical Activity Assessment (0 = self-reported, 1 = objective); Intervention Length (0 = <12 weeks, 1 = ≥12 weeks) Reporting of: Attrition (0 = no, 1 = yes); Compliance (0 = no, 1 = yes); Baseline Physical Activity (0 = no, 1 = yes) Attrition/Compliance: Attrition (0 = >20%, 1 = ≤20%); Compliance (0 = <80%, 1 = ≥80%) Quality Score: 0-4 (Low), 5-7 (Medium), and 8-10 (High).

**References**

1. Arvidsson D, Slinde F, Hulthen L, Sunnegardh J 2009 Physical activity, sports participation and aerobic fitness in children who have undergone surgery for congenital heart defects. Acta Paediatr 98:1475-1482.

2. Barbour-Tuck E, Boyes NG, Tomczak CR, Lahti DS, Baril CL, Pockett C, Runalls S, Kakadekar A, Pharis S, Bradley TJ, Wright KD, Erlandson MC 2020 A cardiovascular disease risk factor in children with congenital heart disease: unmasking elevated waist circumference - a CHAMPS* study *CHAMPS: Children’s Healthy-Heart Activity Monitoring Program in Saskatchewan. BMC Cardiovascular Disorders 20:231.

3. Brudy L, Hock J, Hacker AL, Meyer M, Oberhoffer R, Hager A, Ewert P, Muller J 2019 Children with Congenital Heart Disease Are Active but Need to Keep Moving: A Cross-Sectional Study Using Wrist-Worn Physical Activity Trackers. J Pediatr.

4. Casey FA, Craig BG, Mulholland HC 1994 Quality of life in surgically palliated complex congenital heart disease. Arch Dis Child 70:382-386.

5. Chen CW, Chen YC, Chen MY, Wang JK, Su WJ, Wang HL 2007 Health-promoting behavior of adolescents with congenital heart disease. J Adolesc Health 41:602-609.

6. Ewalt LA, Danduran MJ, Strath SJ, Moerchen V, Swartz AM 2012 Objectively assessed physical activity and sedentary behaviour does not differ between children and adolescents with and without a congenital heart defect: a pilot examination. Cardiol Young 22:34-41.

7. Fredriksen PM, Ingjer E, Thaulow E 2000 Physical activity in children and adolescents with congenital heart disease. Aspects of measurements with an activity monitor. Cardiology in the Young 10:98-106.

8. Hartel JA, Herberg U, Jung T, Winkler C, Breuer J, Muller N 2020 Physical activity and heart rate monitoring in Fontan patients - Should we recommend activities in higher intensities? PLoS One 15:e0228255.

9. Hedlund ER, Lundell B, Villard L, Sjoberg G 2016 Reduced physical exercise and health-related quality of life after Fontan palliation. Acta Paediatr 105:1322-1328.

10. Kao CC, Chang PC, Chiu CW, Wu LP, Tsai JC 2009 Physical activity levels of school-age children with congenital heart disease in Taiwan. Appl Nurs Res 22:191-197.

11. Leitch CA, Karn CA, Ensing GJ, Denne SC 2000 Energy expenditure after surgical repair in children with cyanotic congenital heart disease. J Pediatr 137:381-385.

12. Lunt D, Briffa T, Briffa NK, Ramsay J 2003 Physical activity levels of adolescents with congenital heart disease. Australian Journal of Physiotherapy 49:43-50.

13. Massin MM, Hovels-Gurich HH, Gerard P, Seghaye MC 2006 Physical activity patterns of children after neonatal arterial switch operation. Ann Thorac Surg 81:665-670.

14. McCrindle BW, Williams RV, Mital S, Clark BJ, Russell JL, Klein G, Eisenmann JC 2007 Physical activity levels in children and adolescents are reduced after the Fontan procedure, independent of exercise capacity, and are associated with lower perceived general health. Arch Dis Child 92:509-514.

15. Moschovi D, Kapetanakis EI, Sfyridis PG, Rammos S, Mavrikaki E 2019 Physical activity levels and self-efficacy of Greek children with congenital heart disease compared to their healthy peers. Hellenic J Cardiol.

16. Ray TD, Henry K 2011 Self-efficacy and physical activity in children with congenital heart disease: is there a relationship? J Spec Pediatr Nurs 16:105-112.

17. Ray TD, Green A, Henry K 2011 Physical activity and obesity in children with congenital cardiac disease. Cardiol Young 21:603-607.

18. Stone N, Obeid J, Dillenburg R, Milenkovic J, MacDonald MJ, Timmons BW 2015 Objectively measured physical activity levels of young children with congenital heart disease. Cardiol Young 25:520-525.

19. Voss C, Duncombe SL, Dean PH, de Souza AM, Harris KC 2017 Physical Activity and Sedentary Behavior in Children With Congenital Heart Disease. J Am Heart Assoc 6.

20. Dulfer K, Duppen N, Blom NA, van Dijk AP, Helbing WA, Verhulst FC, Utens EM 2014 Effect of exercise training on sports enjoyment and leisure-time spending in adolescents with complex congenital heart disease: the moderating effect of health behavior and disease knowledge. Congenit Heart Dis 9:415-423.

21. Duppen N, Etnel JR, Spaans L, Takken T, van den Berg-Emons RJ, Boersma E, Schokking M, Dulfer K, Utens EM, Helbing W, Hopman MT 2015 Does exercise training improve cardiopulmonary fitness and daily physical activity in children and young adults with corrected tetralogy of Fallot or Fontan circulation? A randomized controlled trial. Am Heart J 170:606-614.

22. Fredriksen PM, Kahrs N, Blaasvaer S, Sigurdsen E, Gundersen O, Roeksund O, Norgaand G, Vik JT, Soerbye O, Ingjer E, Thaulow E 2000 Effect of physical training in children and adolescents with congenital heart disease. Cardiol Young 10:107-114.

23. Hedlund ER, Lundell B, Soderstrom L, Sjoberg G 2018 Can endurance training improve physical capacity and quality of life in young Fontan patients? Cardiol Young 28:438-446.

24. Klausen SH, Andersen LL, Sondergaard L, Jakobsen JC, Zoffmann V, Dideriksen K, Kruse A, Mikkelsen UR, Wetterslev J 2016 Effects of eHealth physical activity encouragement in adolescents with complex congenital heart disease: The PReVaiL randomized clinical trial. Int J Cardiol 221:1100-1106.

25. Longmuir PE, Tyrrell PN, Corey M, Faulkner G, Russell JL, McCrindle BW 2013 Home-based rehabilitation enhances daily physical activity and motor skill in children who have undergone the Fontan procedure. Pediatr Cardiol 34:1130-1151.

26. Moons P, Barrea C, Suys B, Ovaert C, Boshoff D, Eyskens B, Vandenrijn C, Sluysmans T 2006 Improved perceived health status persists three months after a special sports camp for children with congenital heart disease. Eur J Pediatr 165:767-772.

27. Morrison ML, Sands AJ, McCusker CG, McKeown PP, McMahon M, Gordon J, Grant B, Craig BG, Casey FA 2013 Exercise training improves activity in adolescents with congenital heart disease. Heart 99:1122-1128.

28. Rhodes J, Curran TJ, Camil L, Rabideau N, Fulton DR, Gauthier NS, Gauvreau K, Jenkins KJ 2006 Sustained effects of cardiac rehabilitation in children with serious congenital heart disease. Pediatrics 118:e586-593.
